# Molecular retargeting of antibodies converts immune defense against oncolytic viruses into cancer immunotherapy

**DOI:** 10.1038/s41467-019-11137-5

**Published:** 2019-07-19

**Authors:** Julia Niemann, Norman Woller, Jennifer Brooks, Bettina Fleischmann-Mundt, Nikolas T. Martin, Arnold Kloos, Sarah Knocke, Amanda M. Ernst, Michael P. Manns, Stefan Kubicka, Thomas C. Wirth, Rita Gerardy-Schahn, Florian Kühnel

**Affiliations:** 1 Department of Gastroenterology, Hepatology and Endocrinology, Medical School Hannover, Carl Neuberg Str. 1, 30625 Hannover, Germany; 2 Institute for Clinical Biochemistry, Medical School Hannover, Carl Neuberg Str. 1, 30625 Hannover, Germany; 3 Centre for Innovative Cancer Research, Ottawa Hospital Research Institute, Ottawa, K1H 8L6 Canada; 4 Department of Experimental Hemato-Oncology, Hannover Medical School, Carl Neuberg Str. 1, 30625 Hannover, Germany; 5 Cancer Center Reutlingen, District Hospital, Reutlingen, Germany

**Keywords:** Cancer, Immunology, Molecular medicine, Oncology

## Abstract

Virus-neutralizing antibodies are a severe obstacle in oncolytic virotherapy. Here, we present a strategy to convert this unfavorable immune response into an anticancer immunotherapy via molecular retargeting. Application of a bifunctional adapter harboring a tumor-specific ligand and the adenovirus hexon domain DE1 for engaging antiadenoviral antibodies, attenuates tumor growth and prolongs survival in adenovirus-immunized mice. The therapeutic benefit achieved by tumor retargeting of antiviral antibodies is largely due to NK cell-mediated triggering of tumor-directed CD8 T-cells. We further demonstrate that antibody-retargeting (Ab-retargeting) is a feasible method to sensitize tumors to PD-1 immune checkpoint blockade. In therapeutic settings, Ab-retargeting greatly improves the outcome of intratumor application of an oncolytic adenovirus and facilitates long-term survival in treated animals when combined with PD-1 checkpoint inhibition. Tumor-directed retargeting of preexisting or virotherapy-induced antiviral antibodies therefore represents a promising strategy to fully exploit the immunotherapeutic potential of oncolytic virotherapy and checkpoint inhibition.

## Abstract (web summary)

The efficacy of oncolytic adenoviruses is limited by strong immune responses being induced against the oncolytic virus itself. Here, the authors generate a bispecific molecule capable of redirecting the adenovirus-specific antibodies to tumour cells and show this induces immune mediated cancer growth inhibition and enhances the therapeutic efficacy of viral oncolysis.

## Introduction

By exploiting characteristic molecular alterations of tumor cells, oncolytic viruses preferentially infect and lyse tumors thereby sparing normal tissue^1^. Oncolytic viruses have multiple means of action when affecting tumor cells e.g., by activation of innate immune cells, releasing inflammatory cytokines and tumor antigens, and modulation of the tumor vasculature^2–4^. Induction of tumor-directed adaptive immune responses has been widely acknowledged as a central mechanism to achieve long-term therapeutic success observable in clinical studies^5,6^. However, strong immune responses are also being induced against the oncolytic virus itself, thus limiting the potential of this promising therapy. Neutralizing antiviral antibodies against oncolytic viruses prevent effective infection and reduction of tumor tissue, preclude their repetitive systemic administration, and compromise their use in patients with preexisting immunity^7–10^. Oncolytic adenoviruses of serotype 5 have been intensively investigated as oncolytic agents, have proven safe in application, and showed therapeutic responses in clinical trials^11–13^. Nevertheless, the prevalence of antiadenoviral antibodies, mostly directed against the capsid proteins fiber, penton, and hexon, is extraordinarily high in humans due to previous infections during childhood^14–16^. It has been shown in a syrian hamster model that antitumor activity of oncolytic adenoviruses decrease in the presence of neutralizing antibodies^17^. To circumvent vector neutralization by antibodies, multiple strategies have been pursued, such as using alternative serotypes, exchange of hexon, covering critical capsid structures with immunologically inert residues, or even the use of carrier cells as trojan horses^18–21^.

However, antibodies can also be used as potent anticancer tools. Recombinant monoclonal antibodies recognizing target molecules involved in growth factor signaling and carcinogenesis belong to the indispensable repertoire of clinical tumor therapy. Antibodies such as cetuximab or trastuzumab, directed against EGFR or HER2/neu, respectively, block growth signaling thereby arresting tumor cell proliferation or even inducing apoptosis. When bound to the surface of tumor cells, they may also recruit effector cells such as NK cells, monocytes, and macrophages via their Fc-termini to exert tumor-directed cytotoxicity in a process referred to as antibody-dependent cell-mediated cytotoxicity (ADCC) or, when complement is also engaged, as antibody-mediated complement-dependent cytotoxicity (CDC)^22–24^.

Compared with laboratory-borne monoclonal antibodies for clinical tumor therapy, it can be assumed that antibodies raised by viral infections, including those raised by an oncolytic virus infection, are optimally adapted to cope best with the pathogenic threat. We therefore hypothesized that if this effective humoral response elicited by an oncolytic virus could be redirected against the tumor cell surface, one of the most limiting side effects of virotherapy would be converted into a tumor-directed immunotherapeutic tool.

In our study, we investigate tumor retargeting of antiviral antibodies by using a recombinant bifunctional adapter protein (DE1scFv-pSia) containing the DE1 domain of the adenoviral hexon for capturing adenoviral-specific antibodies and a polysialic acid-specific scFv for tumor cell recognition. Intravenous administration of this adapter in adenovirus-immunized mice effectively inhibits the growth of subcutaneous tumors and prolongs survival by engagement of NK and CD8 T cells. Finally, retargeting of antiviral antibodies significantly amplifies the therapeutic effect of virotherapy and proves a feasible method to immunoactivate tumors for subsequent PD-1/PD-L1 checkpoint inhibition thus facilitating long-term survival.

## Results

### A bifunctional adapter for retargeting of antiviral antibodies

**Fig. 1:**
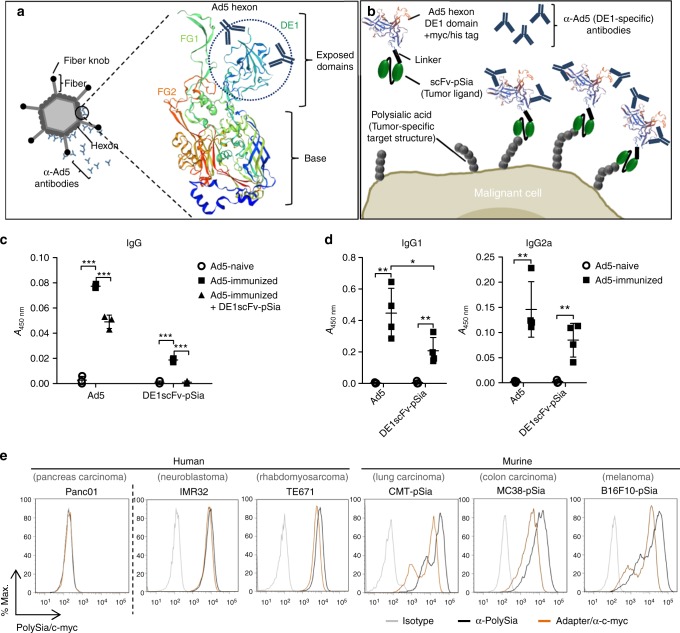
Design of the bifunctional adapter DE1scFv-pSia for tumor retargeting of antiadenoviral antibodies. **a** Illustration of the adenovirus capsid protein hexon generated by ExPASy software using the SWISS-Model tool^68,69^ showing the exposed domains DE1, FG1 and FG2. The domain DE1 is highlighted by a dotted circle **b** The structure of the adapter molecule DE1scFv-pSia containing the DE1 domain (DE1) provided with a myc/his tag which was linked to an anti-PolySia scFv fragment via a glycine/serine stretch (Linker). **c** ELISA showing the recognition of purified, immobilized DE1scFv-pSia by IgG in serum of Ad5-naive, or in serum of Ad5-immunized mice pretreated with or without soluble DE1scFv-pSia as competitor (group: Ad5^+^ + DE1scFv-pSia) to inactivate DE1-specific IgG (*n* = 3). **d** Recognition of immobilized DE1scFv-pSia or Ad5-particles by IgG1 and IgG2a in serum of Ad5-naive (*n* = 3) and Ad5-immunized (Ad5^+^; *n* = 4) mice was measured by ELISA. **e** Binding of DE1scFv-pSia to the polySia-positive human cancer cell lines IMR32 and TE671 and the murine polySia-expressing cancer cells CMT-pSia, MC38-pSia, and B16F10-pSia was measured via flow cytometry. polySia-negative human Panc01 cells were used as negative control. Binding of DE1scFv-pSia to the cell surface was detected using an anti-myc-tag antibody. PolySia expression on the cell surface was measured using the specific antibody mAb735. Two-tailed unpaired *t* test was used to calculate statistics in **c** and **d**: *p ≤ 0.05; **p ≤ 0.01; ***p ≤ 0.001. Error bars refer to standard deviation (SD). Source data are provided as a source data file

First, we generated a bispecific molecule capable of redirecting a substantial proportion of adenovirus-specific antibodies to tumor cells. A preferred target of neutralizing antibodies in adenovirus infections is the hexon protein, the major constituent of the adenoviral capsid. Within the hexon protein, the domains DE1, FG1, and FG2 are externally exposed^25^, and contain important epitopes that are recognized by neutralizing antibodies^15,26,27^ as illustrated in Fig. 1a. Sequence substitutions within these loop domains are sufficient to circumvent antibody neutralization in adenovirus-immunized mice^28^. For our experiments, we chose the hexon DE1 domain for the generation of a bispecific adapter to allow for binding of a significant amount of Ad5-specific antibodies. In preliminary experiments, we found that the isolated DE1 domain could be conveniently prepared in soluble form (data not shown). To facilitate effective recognition of tumor cells for tumor-specific retargeting of DE1-bound antiviral antibodies, we fused DE1 to a previously described single-chain variable fragment recognizing polysialic acid (polySia) ^29^, resulting in the adapter molecule DE1scFv-pSia. The concept of molecular retargeting of antiadenovirus antibodies to tumor cells (further referred to as Ab-retargeting) is illustrated in Fig. 1b. In adults, expression of polySia is highly tumor selective and is abundant on clinically relevant tumors, including small and non-small cell lung cancer, rhabdomyosarcoma, or glioblastoma^30,31^.

First, we examined the capability of DE1scFv-pSia to selectively bind adenovirus-specific immunoglobulins, particularly IgG as the predominant source of antiviral, neutralizing antibodies. We obtained serum of naive and adenovirus-immunized mice for ELISA assays using either immobilized DE1scFv-pSia or complete virus capsids as targets (Fig. 1c). The results show that DE1scFv-pSia-specific antibody activity in serum of Ad5-immunized mice was ~25% of antibody activity directed against complete viral capsids. Depletion of DE1-specific IgGs from serum of Ad5-immunized mice by pre-incubating with soluble DE1scFv-pSia resulted in a significant drop of total antiadenoviral virus-binding IgGs and a complete loss of antibody activity against DE1. These data confirm that DE1scFv-pSia was capable of binding a significant proportion of all virus-specific IgGs (Fig. 1c). As shown in Fig. 1d, the observed DE1-specific antiviral antibodies included at least IgG1 and IgG2a subclasses which are known to induce ADCC, but vary with regard to their ability to activate different effector cells^32^. These findings show the capacity of hexon DE1 to bind a substantial proportion of total virus-directed antibodies that could be sufficient for achieving a therapeutic benefit once redirected in a tumor-specific manner.

Next, we confirmed the function of the used scFv domain by assessing the binding to polySia-expressing cells. Selective binding of DE1scFv-pSia to human IMR32 and TE671 tumor cells (neublastoma and rhabdomyosarcoma, respectively) which intrinsically express polySia, in contrast to polySia-negative Panc01 cells as a negative control, was shown by flow cytometry (Fig. 1e, left panel), confirming that the DE1 domain did not compromise the function of the linked scFv. Since suitable tumor cell lines for syngeneic tumor models in C57BL/6 mice with endogenous expression of polySia are not yet available, we generated transgenic cell lines as described in the Methods section. A particular advantage of this approach is the selection of cell lines syngeneic for the C57BL/6 background which have been well characterized in tumor immunological studies. Those include MC38 colon carcinoma, B16F10 melanoma cells, and CMT-64 non-small cell lung cancer cells, whose neoantigenomes have been recently deciphered thus facilitating the analysis of neoepitope-specific CD8 T-cell responses^33–35^. Using the resulting transgenic derivatives B16F10-pSia, MC38-pSia, and CMT-pSia, we determined binding of DE1scFv-pSia (Fig. 1e, right panel) to the surface of these cells. The analysis confirmed that polySia levels on the surface of these transgenic cell lines were equivalent to human cell lines with endogenous polySia levels. These analyses verified the bispecific properties of DE1scFv-pSia that should facilitate effective retargeting of virus-specific antibodies to polySia-expressing target cells.

### Ab-retargeting inhibits tumor growth and improves survival

**Fig. 2:**
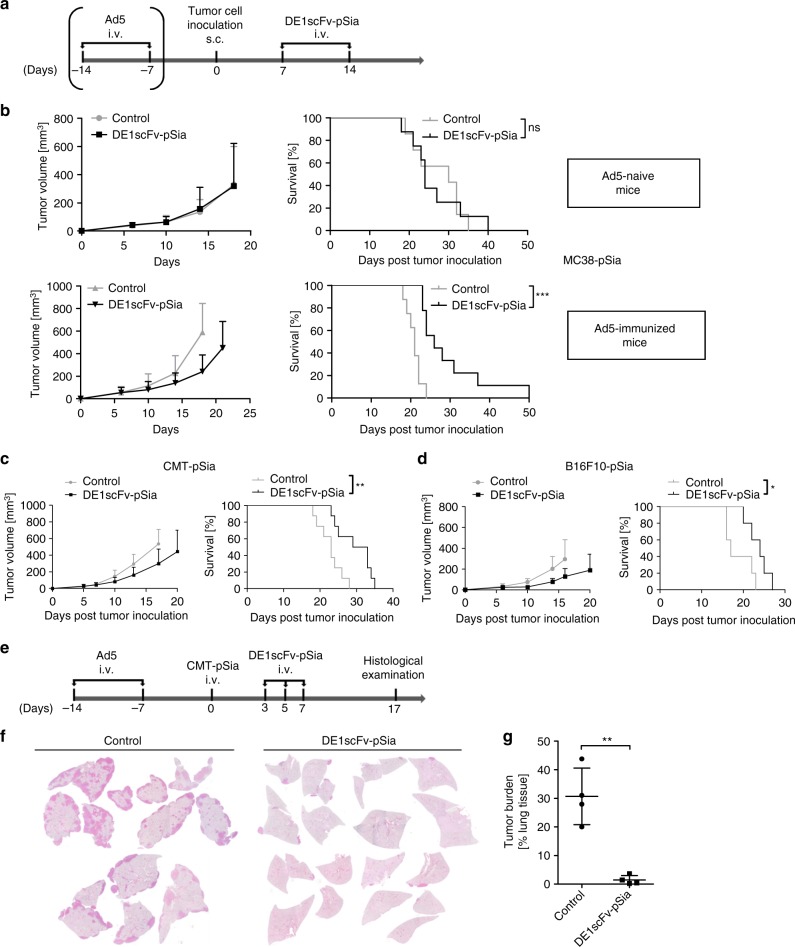
Ab-retargeting inhibits the growth of subcutaneous and disseminated tumors in Ad5-vaccinated mice. **a** Illustration showing the experimental time course of tumor establishement in Ad5-immunized or Ad5-naive mice by s.c. injection of PolySia-expressing tumor cells (MC38-pSia, ‚CMT-pSia, or B16F10-pSia as indicated in the Fig. 2b–d) and subsequent treatments with purified DE1scFv-pSia, or saline, respectively. **b** MC38-pSia tumors were established in Ad5-naive mice (upper panel) or Ad5-immunized mice (lower panel), and treated with DE1scFv-pSia or saline (control). Tumor development (left panel) and survival (right panel) were monitored. Group size: Ad5-naive control, *n* = 7; Ad5-naive DE1scFv-pSia treated, *n* = 8; Ad5-immunized control, *n* = 8; Ad5-immunized DE1scFv-pSia treated, *n* = 9. Median survival (ms) in days for Ad5-naive control: 30; Ad5-naive DE1scFv-pSia treated: 24; Ad5-immunized control: 21; Ad5-immunized DE1scFv-pSia treated: 26. **c** Tumor development (left panel) and survival (right panel) were monitored in Ad5-immunized mice bearing CMT-pSia tumors. Group size: *n* = 8; ms control: 23; ms DE1scFv-pSia: 31. **d** Tumor development (left panel) and survival (right panel) were monitored in Ad5-immunized mice B16F10-pSia tumor-bearing mice. Group size: *n* = 5; ms control: 17; ms DE1scFv-pSia: 24. **e** According to the shown treatment scheme, Ad5-immunized mice were injected i.v. with the lung adenocarcinoma cell line CMT-pSia to establish lung colonies. Mice were then treated i.v. with purified DE1scFv-pSia at the indicated time points or received saline as control. **f** Representative H/E-stained lung sections of control and DE1scFv-pSia treated individuals at endpoint examination (day 17). **g** Tumor burden in the lung was calculated based on microscopic investigations of H/E-stained lung sections (n = 4). Log-rank (Mantel–Cox) test was used to calculate survival in **b**, **c**, and **d** and two-tailed unpaired *t* test was used to calculate statistics in **g**: *p ≤ 0.05; **p ≤ 0.01, ***p ≤ 0.001. Error bars refer to standard deviation (SD). Source data are provided as a source data file

First, we investigated the capability of DE1scFv-pSia-mediated Ab-retargeting to interfere with tumor progression in proof-of-principle experiments. For this purpose, we tested Ab-retargeting in a syngeneic model of subcutaneous MC38-pSia tumors established on C57BL/6 mice. Prior to tumor establishment, mice were either immunized by a double-systemic Ad5 infection to develop antiadenoviral antibodies or were left untreated as naive controls. Purified DE1scFv-pSia was administered twice according to the scheme shown in Fig. 2a. Tumor growth as well as survival was monitored (Fig. 2b). In adenovirus-immunized mice, DE1scFv-pSia administration resulted in delayed tumor growth and significantly prolonged survival when compared with the saline-treated control mice. Since the adapter had no effect in naive mice, these observations demonstrate that preexisting antiadenoviral immunity was essential for antitumor activity in vivo. The results show that inhibition of tumor growth was not caused by the bispecific adapter protein itself. The results additionally suggest that no substantial antibody response has been induced by the viral antigen present in the adapter molecule. We also investigated the antitumor effect of Ab-retargeting in two additional models. The treatment scheme shown in Fig. 2a was applied in adenovirus-preimmunized mice with subcutaneous CMT-pSia or B16F10-pSia tumors, respectively. In both models, tumor growth was inhibited resulting in significantly improved survival (Fig. 2c, d) thus confirming the therapeutic activity of Ab-retargeting. To provide further proof that both antibody engagement and the tumor-binding function are essentially involved in the antitumor effect by Ab-retargeting, we used preimmunized and naive mice to monitor the subcutaneous growth of CMT-pSia cells stably expressing the adapter DE1scFv-pSia, or a soluble DE1 domain lacking the tumor-binding ligand. Tumor growth was only inhibited in the presence of the functional adapter protein and preexisting antiadenoviral immunity (Supplementary Fig. 1). Consistent with the observation that intravenous adapter application did not affect tumor growth in the adenovirus–naive MC38 model, these data provide additional proof that Ab-retargeting depends on tumor-specific retargeting of preexisting antiadenoviral antibodies.

Due to the fact that antibodies can effectively penetrate tissues, we reasoned that Ab-retargeting could be a suitable tool for the treatment of disseminated disease. To investigate this, we analyzed the efficacy of DE1scFv-pSia treatment when applied to Ad5-immunized mice that had been inoculated with CMT-pSia cells by intravenous instillation (Fig. 2e) according to a model of aggressively growing lung colonies as described previously^29^. Repeated intravenous administrations of DE1scFv-pSia led to an almost complete elimination of the tumor burden in the lungs of treated animals (Fig. 2f, g). This result suggests that the established Ab-retargeting is applicable for the treatment of disseminated cancers, and is particularly potent against the formation and outgrowth of metastases. In summary, the findings described above show that DE1scFv-pSia allows tumor retargeting of adenovirus-specific antibodies in vivo and represents an effective antitumor treatment.

### Ab-retargeting activates the tumor microenvironment

**Fig. 3:**
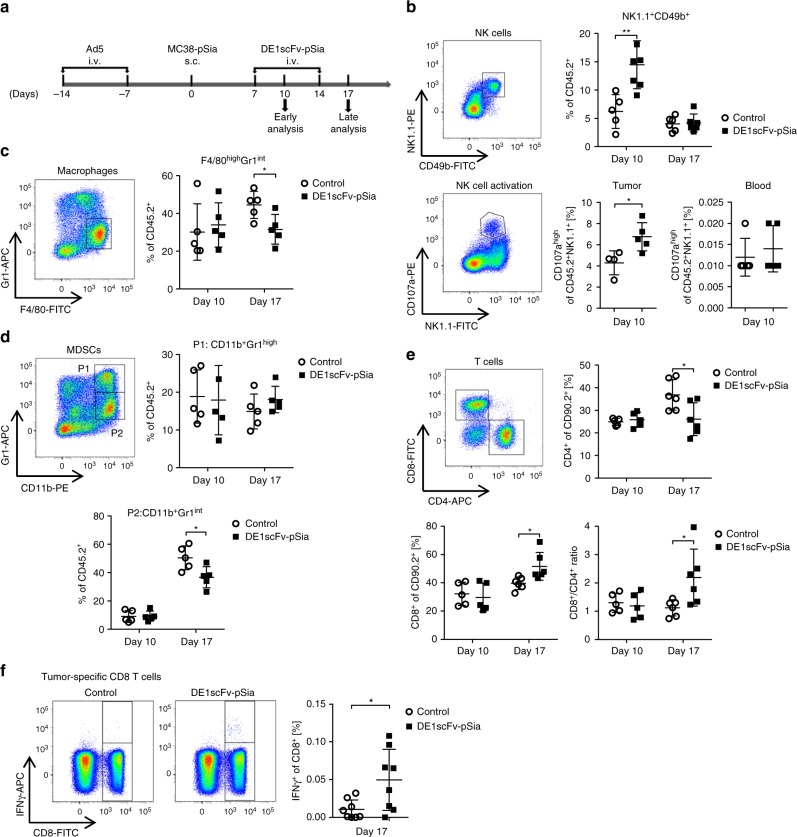
Ab-retargeting reduces intratumor myeloid cells and supports NK cell and CD8 T-cell infiltration. **a** MC38-pSia cells were used to establish s.c. tumors in Ad5-vaccinated mice. Tumor-bearing mice received i.v. injections of DE1scFv-pSia or saline, and were sacrificed according to the shown schedule. Tumor tissue was examined for infiltration of different leukocyte subsets via flow cytometry. **b** Frequencies of NK cells (NK1.1^+^CD49b^+^) were calculated as percentage of CD45.2^+^ leukocytes. NK cell activation was determined by surface expression of CD107a. Macrophages (Gr1^+^F4/80^high^) and different fractions of myeloid-derived suppressor cells (MDSCs; P1: CD11b^+^ Gr1^high^; P2: CD11b^+^ Gr1^int^) are shown in **c** and **d**, respectively. Group size was in general *n* = 5 with following exceptions: *n* = 7 (NK cells/day 10/DE1scFv-pSia); *n* = 6 (NK cells/day 17/control and DE1scFv-pSia groups); *n* = 4 (CD107a^high^ – NK cells/control). **e** Frequencies of CD4^+^ and CD8^+^ T cells were measured as percentage of CD90.2-positive lymphocytes, and individual ratios of CD8 to CD4 T cells were calculated. Group size: n = 5 (day 10) and *n* = 6 (day 17). **f** Splenocytes were prepared from sacrificed mice, incubated with the mutated MC38 peptide Adpgk-R304M (ASMTNMELM), or an irrelevant control peptide, and responding neoantigen-reactive CD8 T cells were identified by intracellular staining for IFNγ (group size: *n* = 8). Two-tailed unpaired *t* test was used to calculate statistics: *p ≤ 0.05; **p ≤ 0.01. Error bars refer to standard deviation (SD). Source data are provided as a source data file

After confirming therapeutic activity in several syngeneic tumor models in vivo, we examined the tumor immune cell contexture in the MC38 model at different time points after Ab-retargeting according to the scheme of treatment and analysis shown in Fig. 3a. After binding their cognate targets, antibodies have the potential to directly recruit and activate innate immune cells via their Fab and Fc-domains^36–38^. Therefore, we investigated abundance and molecular characteristics of tumor-infiltrating myeloid cells and NK cells by flow cytometry. Our data show a profound increase of tumor-infiltrating NK cells in MC38 tumors early after starting the DE1scFv-pSia treatment in Ad5-vaccinated mice (Fig. 3b, upper panel), suggesting a therapy-induced NK cell recruitment into the tumor. Along with an increased NK cell frequency after the first adapter treatment, levels of the degranulation marker CD107a were significantly elevated on the surface of NK cells isolated from tumors of treated animals (Fig. 3b, bottom panel) demonstrating that these NK cells were activated^39^. NK cell infiltration and activation specifically occurred in the tumor since we did not observe changes in the number or activation status of NK cells in peripheral blood (Supplementary Fig. 2). In contrast, the frequencies of tumor-infiltrating macrophages as well as CD11b^+^ Gr1^+^ myeloid-derived suppressor cells (MDSC) were not affected early after therapy initiation (Fig. 3c, d). At later time points, the number of macrophages decreased. When analyzing subpopulations of MDSCs with regard to Gr1 expression, we found that the levels of the fraction P1:CD11b^+^ Gr1^high^ were unchanged, whereas a fraction with reduced levels of Gr1 (P2: CD11b^+^ Gr1^int^ cells) was decreased which presumably reflects a monocytic subpopulation that has been associated with significant immunosuppressive properties^40,41^. These data suggest decreased immunosuppression in the tumor microenvironment due to Ab-retargeting. Consistent with lower immunosuppression as an important precondition to promote the induction of tumor-directed T-cell responses, we observed an increase in tumor-infiltrating CD8 T cells 10 days after therapy initiation and a modified CD8/CD4 ratio in favor of CD8 T cells (Fig. 3e). These observations could be confirmed in the CMT-pSia model (Supplementary Fig. 3). The already known spectrum of CD8 T-cell neoepitopes of MC38 cells^33^ enables the measurement of tumor-directed T-cell responses in an antigen-specific manner. To determine the induction of tumor antigen-specific CD8 T cells by Ab-retargeting, we investigated whether DE1scFv-pSia therapy is able to trigger a specific CD8 T-cell response against the mutated neoantigen Adpgk-R304M by intracellular cytokine staining. Consistent with the T-cell analysis above, the result shows a significantly elevated frequency of Adpgk-specific CD8 T cells in splenocytes of DE1scFv-pSia-treated animals compared with control mice receiving saline (Fig. 3f). Together, these results suggest that Ab-retargeting leads to a an immune activation in the tumor microenvironment involving NK cells and myeloid cells, as well as the triggering of tumor-directed CD8 T-cell responses.

### The benefit of Ab-retargeting depends on NK and CD8 T cells

**Fig. 4:**
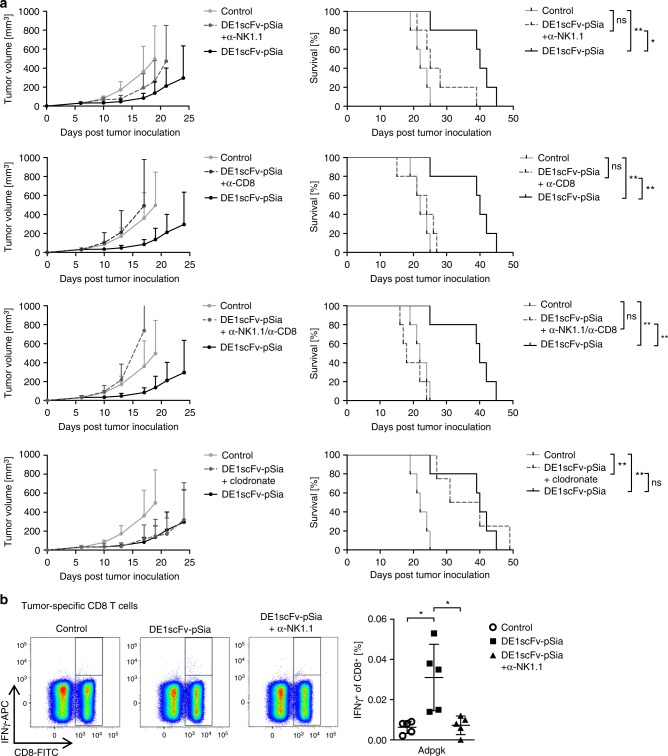
The therapeutic effect of Ab-retargeting is mediated by NK cell-dependent triggering of CD8 T cells. **a** According to the procedure illustrated in Fig. 2a, subcutaneous MC38-pSia tumors were established in Ad5-vaccinated mice and treated with i.v. injections of DE1scFv-pSia or saline. In addition, the influence of NK cells, CD8 T cells, and macrophages on treatment success was studied by depletion of the indicated immune cell subsets starting 2 days before first adapter treatment using depleting antibodies α-NK1.1 and α-CD8 for depletions of NK cells and CD8 T cells, respectively, or clodronate liposomes for depletion of macrophages. Tumor development (left) and survival (right) were monitored. Group size *n* = 5 for all groups, except for the macrophage depleted group (DE1scFv-pSia + clodronate; *n* = 4); the same control and DE1scFv-pSia group without depletion agent is shown in each plot. **b** On day 17 after tumor inoculation, splenocytes of control and adapter-treated groups with or without NK cell depletion were prepared and examined for Adpgk-R304M-reactive tumor-specific CD8 T cells (*n* = 5 per group) by incubation with the corresponding peptide ASMTNMELM as described in Fig. 3f. Log-rank (Mantel–Cox) test was used to calculate survival statistics in **a**. Two-tailed unpaired *t* test was used to calculate statistics in **b**. *p ≤ 0.05; **p ≤ 0.01. Error bars refer to standard deviation (SD). Source data are provided as a source data file

The observation of early NK cell activation in the TME, followed by reduced immunosuppression by macrophages and MDSCs, and a later shift toward CD8 T-cell responses, suggest that these cells may contribute to the therapeutic effect of Ab-retargeting. To address a functional involvement of these cell types in vivo, we depleted NK cells, CD8 T cells, and macrophages during adapter treatment and analyzed the impact of depletion on therapeutic benefit by monitoring tumor progression and survival in the MC38 model (Fig. 4a). It has to be pointed out that the shown subpanels do not reflect independent experiments, since the curves of the depletion groups were separated for better clarity. According to a method reported previously, low dose NK1.1 antibody was administered for selective depletion of NK cells^42^. Macrophage depletion using clodronate did not alter the outcome of therapy, suggesting that these cells were not involved in mediating the therapeutic effect of Ab-retargeting. In contrast, therapeutic efficacy was significantly inhibited after NK cell depletion, indicating a functional role of these cells. Depletion of CD8 T cells almost completely abolished therapeutic efficacy of Ab-retargeting, and no therapeutic effect at all was observed after combined NK and CD8 T-cell depletion. These findings confirm that the antitumor effect strongly depends on both NK cells and CD8 T cells. Previous studies have shown that NK cells essentially contribute to the induction of antigen-specific CD8 T cells by interacting with dendritic cells^43–45^. To investigate such a mutual dependency of NK cell activation and subsequent triggering of tumor-directed CD8 T-cell responses by DE1scFv-pSia-dependent Ab-retargeting, we determined the impact of NK cell deficiency during treatment on the induction of tumor antigen-specific CD8 T cells (Fig. 4b). NK cell depletion completely inhibited the induction of Adpgk-specific CD8 T cells, confirming that effective triggering of tumor-specific CD8 T cells requires the activation of NK cells by Ab-retargeting.

### Ab-retargeting enhances the antitumor effect of virotherapy

**Fig. 5:**
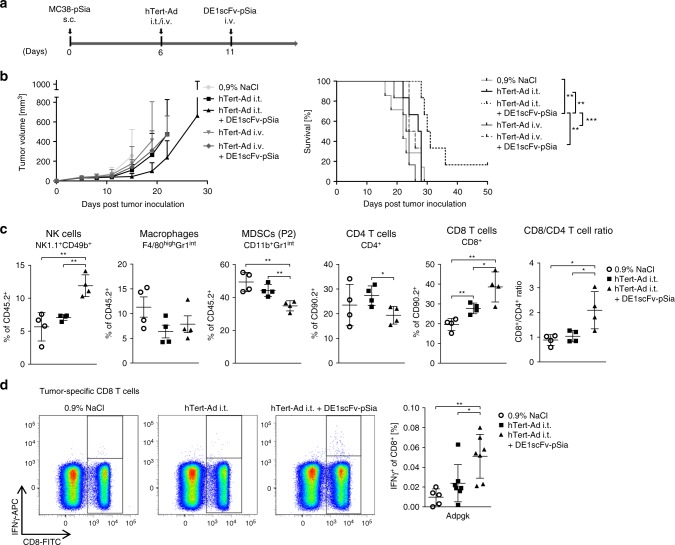
Ab-retargeting after intratumor application of an oncolytic adenovirus leads to improved survival. **a** Subcutaneous MC38-pSia tumors were established in Ad5-naive mice. After tumor formation, animals received a single application of the oncolytic adenovirus hTert-Ad (by either intratumor or intravenous application) followed by a single i.v. administration of DE1scFv-pSia as shown in the treatment scheme. Control animals were treated by i.t. injection of saline. **b** Tumor development and survival were monitored (group size *n* = 6, except control group 0.9 % NaCl: *n* = 7, median survival: 0.9% NaCl 22 days; hTert-Ad i.v. 23.5 days; hTert-Ad i.t. 27.5 days; hTert-Ad i.v. + DE1scFv-pSia 25 days; hTert-Ad i.t. + DE1scFv-pSia 30.5 days). **c** Tumor-infiltrating immune cells were analyzed on day 8 after start of i.t. virotherapy (*n* = 4 in all groups). Proportions of NK cells, macrophages, and MDCSs were calculated as percentage of CD45.2-positive tumor-infiltrating leukocytes. CD8 and CD4 T-cell frequencies were calculated as percentage of CD90.2-positive lymphocytes. **d** Analyses of tumor antigen-specific CD8 T cells in splenocytes 13 days after i.t. virotherapy (0.9% NaCl: *n* = 5; hTert-Ad: *n* = 7; hTert-Ad + DE1scFv-pSia: *n* = 7). To determine neoantigen-specific responses against Adpgk-R304M, splenocytes were stimulated with the peptide ASMTNMELM, or an irrelevant control peptide, and were analyzed by intracellular staining of IFNγ and flow cytometry. Log-rank (Mantel–Cox) test was used to calculate survival statistics in **b**. Two-tailed unpaired *t* test was used to calculate statistics in **c** and **d**. *p ≤ 0.05; **p ≤ 0.01; ***p ≤ 0.001. Error bars refer to standard deviation (SD). Source data are provided as a source data file

In the experiments described above, we used mice which had been systemically immunized with adenovirus to induce humoral responses for subsequent retargeting experiments. According to our initial hypothesis, we wanted to test whether DE1scFv-pSia-mediated Ab-retargeting could be used to exploit virus-directed antibodies that have been collaterally induced by oncolytic adenovirus application in tumor-bearing mice. To investigate the impact of Ab-retargeting as a therapeutic intervention subsequent to virotherapy, we established subcutaneous MC38-pSia tumors in naive mice and treated established tumors by a single intratumor or intravenous injection of the oncolytic adenovirus hTert-Ad^46^ followed by a single DE1scFv-pSia treatment 5 days after virotherapy. The experimental setup is illustrated in Fig. 5a. As shown in Fig. 5b, only a sequential therapy consisting of intratumor oncolytic virotherapy and a subsequent single dose of DE1scFv-pSia inhibited tumor growth, significantly prolonged survival including long-term survival. When the oncolytic adenovirus was applied by intravenous injection prior to adapter application, a slight reduction of tumor growth was observed compared with intravenous injection of the virus alone, but no significant life-prolonging effect was achieved. When applied systemically, oncolytic adenoviruses serotype 5 usually lead to very poor tumor transduction, since the vast majority of the viral load is cleared by the liver^47,48^. This is consistent with the observation in our model that intratumor virus application inhibited tumor growth more effectively compared with intravenous application. Since we had shown in our previous experiments that two subsequent applications of DE1scFv-pSia alone did not facilitate complete regression in any case (Fig. 2b), our observations therefore suggest that these two immunotherapeutic interventions act in a complementary fashion that may lead to long-term tumor-free survival. To further characterize the immune mechanisms triggered by combined therapy, we quantified tumor-infiltrating immune cells after intratumor virotherapy alone compared with virotherapy and Ab-retargeting (Fig. 5c). The results show enhanced tumor infiltration by NK cells and a reduced frequency of monocytic MDSCs in the combination treatment compared with virotherapy alone. Macrophages were reduced in all treatment groups. T-cell responses significantly shifted toward a CD8 T-cell response after sequential therapy compared with virotherapy alone. Accordingly, analyses of tumor-specific CD8 T cells against the MC38 neoantigen Adpgk in splenocytes showed a significant increase of these antitumor T cells after combination therapy (Fig. 5d). These data demonstrate that Ab-retargeting can be actually applied to exploit virotherapy-induced humoral immune responses, and is capable of significantly improving the outcome of virotherapy. These data also suggest that a coordinated combination of these two immunotherapies synergize to induce an immune activation at the site of the tumor, subsequently facilitate the induction of tumor-reactive CD8 T cells, and achieve an overall therapeutic benefit compared with virotherapy alone.

### Ab-retargeting sensitizes the tumor for checkpoint inhibitors

**Fig. 6:**
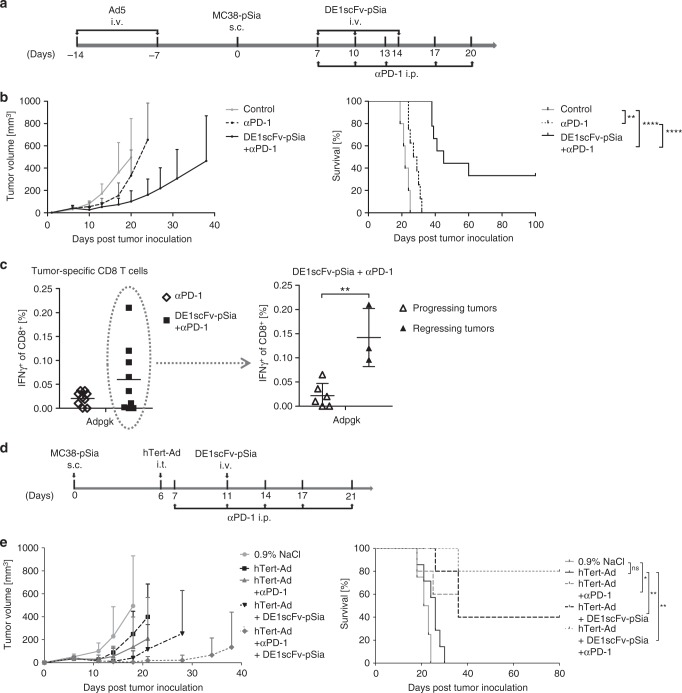
Ab-retargeting sensitizes tumors for PD-1 checkpoint inhibition facilitating long-term survival. **a** Subcutaneous MC38-pSia tumors were established in Ad5-vaccinated mice. Seven days after tumor cell injection, animals were treated with an antagonistic antibody against PD-1 as monotherapy (αPD-1) or in combination with DE1scFv-pSia antibody retargeting (DE1scFv-pSia + αPD-1) or remained untreated (control). **b** Tumor development and survival (group size: αPD-1: *n* = 8, ms: 28 days; DE1scFv-pSia + αPD-1: *n* = 9, ms: 45 days, control: *n* = 5, ms: 22 days) was monitored. **c** Blood samples were drawn during treatment on day 13 after start of treatment, and splenocytes were stimulated with the peptide ASMTNMELM to detect CD8 T-cell responses against the Adpgk-R304M neoepitope. Responsiveness of CD8 T cells to peptide stimulation was measured via intracellular IFNγ-staining and flow cytometry. Left panel: αPD-1 vs. DE1scFv-pSia + αPD-1 (*n* = 9 per group). Right panel: individuals of the DE1scFv-pSia + αPD-1 treatment group were split in those showing tumor progression (*n* = 6) and tumor-free animals: (*n* = 3). **d** Subcutaneous MC38-pSia tumors were established in Ad5-naive mice. Tumor-bearing mice were treated by i.t. application of hTert-Ad as monotherapy with or without i.p. PD-1 immune checkpoint inhibition, and/or antibody-retargeting according to the treatment scheme. **e** The left panel shows the results of tumor growth monitoring and the right panel survival data (group size *n* = 5, except the groups 0.9 % NaCl: *n* = 4 and hTert-Ad: *n* = 7; median survival: 0.9% NaCl ms = 22; hTert-Ad: ms = 26; hTert-Ad + αPD-1: ms = 36; hTert-Ad + DE1scFv-pSia: ms = 36; hTert-Ad + αPD-1 + DE1scFv-pSia ms = undefined). Log-rank (Mantel–Cox) test was used for survival statistics in **b** and **d**. Two-tailed unpaired *t* test was used for statistics in **c**. *p ≤ 0.05; **p ≤ 0.01; ****p ≤ 0.0001. Error bars refer to standard deviation (SD). Source data are provided as a source data file

Cancer treatments with immune checkpoint inhibitors targeting CTLA-4 or the PD-1/PD-L1 axis can prevent dysfunction of tumor-directed CD8 T-cell responses and have shown durable responses in humans with advanced cancers^49^. Unfortunately, only a small proportion of tumors actually responds to these therapies, whereas the majority remains therapy-resistant. Since tumor infiltration by immune cells, particularly CD8 T cells, has been associated with an improved response to checkpoint inhibitors and overall survival^50^, we intended to investigate whether Ab-retargeting could serve as a suitable primary intervention to activate tumors for subsequent immune checkpoint inhibition. To this end, we used a PD-1 antagonistic antibody in Ad5-vaccinated mice bearing subcutaneous MC38-pSia tumors pretreated with DE1scFv-pSia as illustrated in Fig. 6a. Responsiveness of MC38-pSia to PD-1 monotherapy was limited, and only led to a transient growth inhibition with a negligible impact on survival (Fig. 6b). In contrast, retargeting of these preexisting antibodies by DE1scFv-pSia administrations in parallel to PD-1 checkpoint inhibition led to an improved inhibition of tumor growth and a significantly prolonged survival, including complete tumor remissions in three of nine treated individuals. To investigate whether improved therapeutic outcome was associated with increased frequencies of tumor-directed CD8 T cells, we examined blood samples of animals treated with either αPD-1 alone or the combination of αPD-1 and DE1scFv-pSia (Fig. 6c). Analysis of CD8 T cells specific for Adpgk confirmed a visible increase of these tumor-directed CD8 T cells following combined treatment in comparison with αPD-1 treatment alone (Fig. 6c, left panel). In the group that received the combined treatment, we distinguished between animals that had progressing tumors and animals with complete tumor remissions to investigate whether the frequency of Adpgk-specific CD8 T cells is indeed positively correlated with a therapeutic benefit (Fig. 6c, right panel). We observed significantly elevated levels of these neoantigen-directed CD8 T cells in individuals that responded by complete tumor regression when compared with CD8 T-cell numbers close to base line in non-responding individuals, suggesting a positive correlation of CD8 T cells numbers and response to therapy. Retargeting of prevalent antiviral antibodies appears to effectively sensitize the tumor for checkpoint inhibition. We hypothesized that retargeting of antibodies elicited by oncolytic virotherapy represent the most promising approach to foster tumor responsiveness to checkpoint inhibition. Consequently, we investigated the therapeutic outcome when all three immunotherapeutic interventions mentioned above were combined in a rational therapeutic scheme (Fig. 6d, upper panel). In the presented setting, Ad5-naive animals received oncolytic virotherapy by i.t. injections of hTert-Ad, and were subsequently treated with DE1scFv-pSia for retargeting of antiviral antibodies and simultaneous αPD-1 therapy to amplify the elicited tumor-specific T-cell responses. Consistent with our hypothesis, we observed that the majority of treated animals experienced complete and durable tumor remissions (Fig. 6d, bottom panel). These results indicate that virotherapy followed by retargeting of the antiviral antibodies effectively converts tumors to an immunoactivated state accessible for PD-1 checkpoint inhibition.

In summary, our study shows that molecular retargeting of antiviral antibodies against tumors by using bispecific adapter proteins inhibits tumor growth and improves survival in murine tumor models. Furthermore, this study demonstrates that Ab-retargeting is able to convert a disadvantageous side effect of oncolytic virotherapy into an anticancer tool and serves as a promising measure to render tumors responsive to immune checkpoint inhibition.

## Discussion

Adenoviruses have an outstanding potential to activate the immune system when used as vaccines or oncolytic viruses^51^. Recently, a tropism-modified oncolytic adenovirus has shown promising tumor responses in a phase I trial in glioblastoma patients^13^. However, like two sides of the same coin, only virotherapy-triggered tumor-directed immune responses promise therapeutic benefit whereas responses against the viral vector, such as preexisting or therapy-induced antibodies, compromise applicability, and therapeutic efficacy. Consequently, preventing the neutralization of adenovirus-based vaccines or oncolytic adenoviruses by vector-directed antibodies has been subject to intense research for decades^52^. However, a recent report has shown that preexisting immunity to Newcastle Disease Virus even enhanced its immunotherapeutic activity, when the vector was applied intratumorally^53^, shedding a new light on the impact of preexisting antibody responses. As an innovative approach, we aimed to exploit this antiviral antibody reservoir by converting it into a tumor-directed immune response using molecular retargeting. In our study, we utilized the DE1 domain of adenovirus 5 hexon for the generation of a bifunctional adapter molecule (DE1scFv-pSia) linked to an established polysialic acid-specific scFv as a tumor-specific ligand. The adapter demonstrated a significant tumor therapeutic effect when administered in mice with preexisting humoral Ad5-specific immunity either due to Ad5 vaccinations or tumor virotherapy using the oncolytic adenovirus hTert-Ad. Although Ad5-based oncolytic viruses belong to the most frequently investigated oncolytic vector types, their significant immunogenicity and the wide seroprevalence of Ad5-specific antibodies in humans have been strong arguments against their application as vectors for virotherapy. On the other hand, this provides an excellent rationale for using an Ad5-derived antigen for Ab-retargeting. Our results show that the DE1 domain was capable of binding significant amounts of total antiviral antibodies in the serum of Ad5-immunized mice consistent with reports describing that hexon is one of the major targets of neutralizing antibody responses in men and mice^14,27,54^. In general, effective immunity involves diverse effector mechanisms interacting in a synergistic manner to optimally adapt to the needs to fight a pathogenic thread. Consistently, we found that the antivirus humoral response after challenge with adenovirus 5 encompassed various subtypes of immunoglobulins, as demonstrated for IgG1 and IgG2a, providing a promising substrate to link target and effector cells for ADCC. The role of tumor-directed antibodies in controlling tumor growth is not well understood. However, it has been shown in patients and in a rabbit tumor model using the oncolytic vaccinia virus Pexa-Vec that therapeutic effects correlated with antibody-mediated complement-dependent cytotoxicity^55^. Since we did not observe a role of complement in preliminary experiments, we focused on the role of immune cell candidates that are capable of recognizing antibody-coated cells and can exert ADCC. NK cells are essential effector cells in recognizing malignant and virus-infected cells. When attracted by target cells coated with antibodies of suitable subclasses such as IgG1, NK cells expressing the appropriate Fc receptor can execute cell-to-cell cytolysis^36^. In this case, it has been shown that the Fc domain of immunoglobulins is sufficient to bridge NK cells with target cells to induce their killing^37^. Regarding the fact that the antibodies engaged for molecular retargeting in our study originate from a viral infection, we reasoned that the elicited immunoglobulin spectrum should be well adapted to effectively attract and activate NK cells for target cell killing. We observed a profound increase of activated NK cells in the immune cell infiltrates in tumors after Ab-retargeting. Depletion experiments confirmed the essential role of these cells, since selective NK cell depletion abrogated the therapeutic effect. NK cells are also well known to be involved in shaping subsequent adaptive immunity. First, killed tumor cells are an essential source of antigens for cross-presentation. Second, simultaneous interaction of NK cells with dendritic cells or macrophages determines how antigens are presented to trigger T-cell responses^43,45,56,57^. Macrophages as well as MDSCs were also reduced in tumors after Ab-retargeting. Since tumor-associated macrophages and MDSCs have been associated with immunosuppressive properties^58,59^, these decreased levels of myeloid cells suggest lower levels of immunosuppression, which could promote tumor infiltration with functional CD8 T cells^60^. Consistently, we observed a shift in the CD8/CD4 T-cell ratio toward CD8 T cells after Ab-retargeting. In addition, our data demonstrate an increase in CD8 T cells recognizing the tumor-specific neoepitope Adpgk-R304M, thus confirming an activation of tumor-directed immune responses. Our depletion experiments showed important roles not only for NK but also for CD8 T cells. In line with the hypothesis that NK cell-mediated tumor cell killing induced by Ab-retargeting provides the antigen necessary for priming of tumor antigen-specific CD8 T-cell responses, we showed that the response against the neoepitope Adpgk was completely abrogated when NK cells were depleted during Ab-retargeting. These observations confirmed that NK cell-mediated tumor cell killing after Ab-retargeting decisively shapes antitumor CD8 T-cell responses. Recently, activation of NK cells has been proposed as a means to recognize and eliminate micrometastases^61,62^. Consistently, our experiments in a model of lung colonies suggest that Ab-retargeting is effective in fighting disseminated diseases. A highly effective way to induce significant levels of antibodies amenable for retargeting is a preceding virotherapeutic treatment. In our experiments, we observed that Ab-retargeting after intratumor virotherapy led to a significant survival benefit and even facilitated long-term tumor-free survival in some of the treated animals. This outcome was not achieved in our model with vaccination-induced preexisting antibodies, even after repeated dosage of the adapter, suggesting further levels of synergy between virotherapeutic oncolysis and subsequent Ab-retargeting.

Checkpoint inhibitors have been a success story in the treatment of advanced cancer, but the vast majority of tumor patients do not respond to therapy. Activation of the tumor microenvironment and promoting tumor infiltration by T lymphocytes are generally considered of crucial importance to convert therapy-resistant tumors into tumors suceptible to checkpoint inhibitors. Consequently, virotherapy has been shown to be a promising initiation therapy for subsequent checkpoint inhibition in mice^35,63,64^, and is under investigation in clinical studies^65^. Local injection of the oncolytic virus in accessible tumor nodules reflects the current state-of-the-art. However, this still entails considerable clinical intervention in the vast majority of tumor entities. In Ad5-immunized mice, we found that Ab-retargeting combined with PD-1 checkpoint inhibition allowed for long-term survival in a number of treated animals in the MC38 model which is otherwise resistant to PD-1 monotherapy. Our data therefore suggest that molecular retargeting of preexisting antibodies is a feasible method to activate the tumor microenvironment and to improve infiltration by tumor-directed CD8 T cells to facilitate effective therapy using checkpoint inhibitors. In humans, solid tumors usually emerge in patients with advanced age who have presumably been challenged by multiple virus vaccinations and infections throughout their lifespan providing a rich protective pool of antibodies against several viruses, including measles, vaccinia, coxsackievirus, different adenovirus serotypes, and herpes viruses. Corresponding bifunctional adapter proteins harboring a set of dominant epitopes recognized by these antibodies could be an interesting noninvasive method to activate tumors for subsequent checkpoint inhibition. Nevertheless, virotherapy represents a tumor therapeutic tool by itself, and also an effective trigger of defined antiviral antibodies. Consequently, using an integrated therapeutic scheme comprising intratumor virotherapy for oncolysis and antibody induction, followed by a single dose of Ab-retargeting and PD-1 checkpoint inhibition to maintain and amplify antitumor T-cell responses resulted in long-term survival of the majority of treated animals. In contrast to strategies that circumvent preexisting antibodies by viral capsid modification, retargeting of antiviral antibodies is a fundamentally alternative concept providing several additional ways of synergy with oncolytic virotherapy. Withdrawal of antiviral antibodies from the system by Ab-retargeting could enable oncolytic virus administration in patients with preexisting immunity, enhance the efficacy of viral spread, and facilitate repetitive application. As such a complementary approach, retargeting of vector-neutralizing antibodies to physiological “sinks”, e.g., the reticuloendothelial system of the liver, could be considered^48,66,67^. These aspects certainly warrant further investigation.

In summary, we showed that molecular retargeting is an effective method for engaging antiviral antibodies to recognize and fight tumors. Ab-retargeting is an attractive option to activate tumors for systemic immunotherapies, and will be a promising tool to fully exploit the potential of oncolytic virotherapy.

## Methods

### Materials

All materials used in this study are publicly available and can be obtained from the authors upon request.

### Regulatory approvals

All in vivo experiments were performed according to the German guidelines for animal care and use of laboratory animals (TierSchG). The experiments have been approved by the review boards of Hannover Medical School animal facility (ZTL) and the regional legal authorities (LAVES, Oldenburg, Germany).

### Cell lines and culturing

The cell lines HEK293 (CRL-1573), 293T (CRL-3216), PhoenixAMPHO (CRL-3213), Panc01 (CRL-1469), TE671 (CRL-8805), IMR32 (CCL-127), and B16F10 (CRL-6475) were obtained from ATCC. CMT-64 cells (10032301) were obtained from the European Collection of Cell Cultures (ECACC), and MC38 cells were kindly provided by Michael Neumaier, University Mannheim, Germany. MC38 cells used in the study were authenticated by their characteristic growth patterns in cell culture and by verification of mutation-specific CD8 T-cell responses against the neoantigen adpgk-R304M in vivo. TE671 is a cell line listed in the database of commonly misidentified cell lines (ICLAC). Initially claimed as a medulloblastoma line, TE671 are actually rhabdomyosarcoma cells as we have referred to. The cell line has been chosen for comparative purposes for polySia expression levels on human tumor cells. Cells were cultured at 37 °C and 5% CO_2_ in DMEM + Glutamax (Gibco) supplemented with 10% FCS (Gibco) and 100 U/mL streptomycin and 100 mg/mL penicillin (Gibco). All cell lines were tested for mycoplasma contaminations on a regular basis.

Polysialic acid expressing tumor cells CMT-pSia, MC38-pSia, and B16F10-pSia were generated by double retroviral transduction using PQCXI-based retroviral vectors (Addgene) containing an expression cassette for murine polysialyltransferase ST8SiaIV and neomycin resistance, or the neural cell adhesion molecule (NCAM) and puromycin resistance, respectively. Retroviral particles were produced in PhoenixAMPHO cells by lipofectamine transfection of retroviral vector plasmids. Cell culture supernatant, containing infectious retroviral particles, was supplemented with polybrene (final concentration:8 µg/ml, Sigma) and added to the target cells (CMT64/MC38/B16F10). After 48 h, successfully transduced cells were selected by addition of 1 mg/ml G418 (Biochrom) and 1 µg/ml puromycin (Sigma).

### Preparation of the recombinant adapter molecule DE1scFv-pSia

DE1scFv-pSia was purified via His-tag affinity chromatography using Ni-NTA-Agarose (Qiagen). 293T cells were transfected with a pT3-based expression vector for DE1scFv-pSia. Forty-eight hours post transfection, the cell supernatant was collected. Cells were subjected to free/thaw cycles, and cell extracts were obtained by removing cell debris via centrifugation. Cell supernatants and cell extracts were combined and filtered (45 µm) for further purification. Filtrates were supplemented with sodium phosphate buffer pH 8.0 (50 mM), NaCl (150 mM), and Ni-NTA-Agarose beads (Qiagen). Protein binding was carried out overnight in an overhead shaker at 4 °C. Ni-NTA-Agarose beads were pelleted via centrifugation, and washed twice with phosphate buffer containing sodium phosphate pH 8.0 (50 mM), NaCl (150 mM), and Imidazol (1 mM). Elution was performed by adding phosphate buffer containing sodium phosphate pH 8.0 (50 mM), NaCl (150 mM), and L-Histidin (100 mM) and incubation at 4 °C for 4 h in an overhead shaker. The eluate was collected and stored at −80 °C. Protein concentration was determined using Bio-Rad Protein Assay. Binding of DE1scFv-pSia to target cells was determined via FACS analysis using a c-myc antibody (clone: 9E10, Santa Cruz Biotech.).

### Preparation of recombinant adenovirus

The oncolytic adenovirus hTert-Ad^46^ was used for all experiments in this study. Viral particles were produced in HEK293 cells and purified via CsCl gradient centrifugation. Virus stocks were supplemented with 50% glycerol stock solution containing glycerol (50% v/v), mouse serum albumin (0.1% m/v), Tris (pH 8.0; 10 mM), and NaCl (100 mM), and were kept at −20 °C. Virus was dialyzed against dialysis buffer containing Trisbase pH 8.0 (20 mM), NaCl (25 mM), and MgCl_2_ (1.25 mM), and stored at −80 °C until usage.

### In vivo experiments

All mouse experiments were carried out using 6-week-old immunocompetent C57BL/6-J mice. Mice were immunized against adenovirus serotype 5 by intravenous injections of 1 × 10^9^ infectious particles hTert-Ad in 0.9% NaCl two times with a 7 day interval. Subcutaneous (s.c.) syngeneic tumors were established by s.c. injection of 1 × 10^7^ tumor cells (MC38-pSia/CMT-pSia/B16F10-pSia) in PBS in the flank of the mice. Lung colonies were induced by intravenous (i.v.) injection of 3 × 10^5^ CMT-pSia cells in 0.9% NaCl. Virotherapy was applied by intratumor (i.t.) injection of 1 × 10^9^ infectious particles of hTert-Ad. Adapter treatment was applied by i.v. injections of 50 µg of purified DE1scFv-pSia per injection in 0.9% NaCl. For in vivo depletion of CD8 T cells, NK cells, and macrophages, specific depletion antibodies against CD8 (clone: 116–13.1; 75 µg in 0.9% NaCl), NK1.1 (clone: PK136; 25 µg in 0.9% NaCl solution), and clodronate liposomes (200 µl of a 5 mg/ml solution) were injected intraperitoneally (i.p.) twice a week. Anti-PD-1 immune checkpoint inhibition was achieved by i.p. injections of an antagonistic antibody against PD-1 (clone: RMP1-14; 75 µg in 0.9% NaCl solution) twice a week. Blood samples were withdrawn from the vena facialis to a maximum amount of 200 µl once during treatment phase.

### Preparation of single-cell solutions from tissue and blood

Tumor tissue was digested in the RPMI 1640 medium (Gibco) containing DNAse (60 µg/ml), hyaluronidase (0.2 µl/ml), Collagenase IA and IV (0.2 mg/ml each) for 30 min at 37 °C. Required enzymes were obtained from Sigma. Cell solutions were subsequently washed with RPMI and filtered using a 40 -µm cell strainer to obtain single-cell preparations.

Splenocytes were released from spleens by passing through a 40 -µm cell strainer and washed with RPMI 1640 (Gibco). Erythrocytes were lysed by adding 1x EBC lysis buffer (BioLegend) for 5 min at 4 °C, and cells were subsequently washed with RPMI 1640. Cells were resuspended in the RPMI 1640 medium supplemented with 2% FCS, 100 U/mL streptomycin, 100 mg/mL penicillin, 1% MEM with nonessential amino acids (×100 solution, Gibco) ß-mercaptoethanol (50 µM, Sigma), and sodium pyruvate (1 mM, Gibco), and kept at 37 °C and 5% CO_2_ overnight during peptide stimulation.

Erythrocytes were removed from blood samples by adding ×1 EBC lysis buffer (BioLegend) and incubated for 3 min at 4 °C. Cells were subsequently washed with RPMI 1640 (Gibco).

### Analysis of immune cells

Single-cell solutions prepared from tumor tissue, spleen, or blood were analyzed using a FACSCanto II. FACS data were analyzed using FlowJo 7 (Treestar). Different immune cell types were identified using the following conjugated antibodies from BioLegend in a 1:100 dilution: CD90.2-PerCP (30-H12; Cat. 105338), CD45.2-PerCP (104; Cat. 109826), CD8a-FITC (53–6.7; Cat. 100706), CD8a-PE (53–6.7; Cat. 100708), CD4-PE (GK1.5; Cat. 100408), CD4-APC (GK1.5; Cat. 100412), Gr1-APC (RB6–8C5; Cat. 108412), CD11b-PE (M1/70; Cat. 101208), F4/80-FITC (BM8; Cat. 123108), IFNγ-APC (XMG1.2; Cat. 505810), NK1.1-PE (PK136; Cat. 108708), NK1.1-FITC (PK136; Cat. 108706) CD49b-FITC (DX5; Cat. 108906), CD49b-APC (DX5; Cat. 108910), and CD107a-PE (1D4B; Cat. 121612). The following antibodies were used as isotype controls: rat IgG2a (RTK2758; Cat. 400506/400508; isotype ctrl for: CD107a/ F4/80/ CD8); mouse IgG2a (MOPC-173; Cat. 400208/400212 /400250; isotype ctrl for: NK1.1/ CD45.2); rat IgM (RTK2118; Cat. 400806/400810; isotype ctrl. for: CD49b); rat IgG2b (RTK4530; Cat. 400612/400636/400630; isotype ctrl. for: CD11b/ CD4/ Gr1/ CD90.2); rat IgG1 (RTK2071; Cat. 400412; isotype ctrl for: IFNγ). NK cell, macrophage, and myeloide-derived suppressor cell (MDSC) populations were calculated as percentage of all CD45.2-positive leukocytes and identified by the following marker expression profiles: NK cells: NK1.1^+^ and CD49b^+^; macrophages: Gr1^+^ and F4/80^high^; MDSCs: CD11b^+^ and Gr1^high^(P1)/Gr1^int^ (P2). CD8-positive and CD4-positive T-cell populations were calculated as percentage of CD90.2-positive lymphocytes. The gating strategy to identify immune subpopulations is exemplified in Supplementary Fig. 4.

Tumor-reactive CD8 T cells in prepared splenocytes were identified after stimulation with a MC38-derived neoantigen peptide ASMTN**M**ELM (H-2Kb) specific for the mutated neoantigen adpgk-R304M by intracellular staining of IFNy. During stimulation, cytokine secretion was blocked by addition of 1 µg/µl Brefeldin A (BioLegend). As control, cells were stimulated with the irrelevant control peptide SIINFEKL (H-2Kb) derived from ovalbumin (OVA). IFNγ-producing cells were calculated as percentage of CD8 T cells. Peptides were obtained from Proimmune.

### ELISA analysis

ELISA plates (96-well) were coated with either Ad5 (1 × 10^8^ viral particles/well) or purified DE1scFv-pSia (5 µg/well). Plates were then incubated with either serum from blood of Ad5-naive mice, or Ad5-immunized mice, or serum of Ad5-immunized mice pretreated with DE1scFv-pSia to inactivate DE1-specific antibodies as indicated in the figure legend. IgG binding was detected using the following HRP-conjugated secondary antibodies: horse anti-mouse IgG from Cell Signaling Technology (polyclonal; #7076), rat anti-mouse IgG1 from Invitrogen (clone: LO-MG1-2), and rat anti-mouse IgG2a from Invitrogen (clone: LO-MG2a-3). Plates were developed using the BD OptEIA ELISA Kit. The reaction was terminated by addition of 1 mol/l H_3_PO_4_, and absorbance was measured at 450 nm.

### Histology

Lungs were fixed using paraformaldehyde (PFA), paraffin-embedded and lung tissue sections were subjected to haematoxylin/eosin (H/E) staining. The tumor area was calculated as the percentage of the whole lung tissue using Cell software (Olympus).

### Statistical analysis

The data from two treatment groups were statistically analyzed using GraphPad Prism 5. The results were compared with two-tailed unpaired *t* test and survival curves were analyzed by log-rank (Mantel–Cox) test. All measurements used for statistical evaluation were taken from distinct samples. All values shown in the figures are provided as mean ± SD; *p* < 0.05 was considered statistically significant.

### Reporting summary

Further information on research design is available in the Nature Research Reporting Summary linked to this article.

## Supplementary information

Supplementary Information

reporting summary

Source Data

## Data Availability

The source data underlying Figs 1c,d, 2b,c,d,g, 3b-f, 4, 5b-d, and 6b-d and Suppl. Figs 1, 2, and 3b-e are provided as a Source Data file. All the other data supporting the findings of this study are available within the article and its Supplementary Information files and from the corresponding author upon reasonable request. A reporting summary for this article is available as a Supplementary Information file.
