# Supplementary material for: Molecular retargeting of antibodies converts immune defense against oncolytic viruses into cancer immunotherapy
Source: Nat Commun. 2019 Jul 19;10:3236. doi: 10.1038/s41467-019-11137-5 (PMC6642145; doi:10.1038/s41467-019-11137-5)
Supplement: Supplementary file 1 — Supplementary Information [file 41467_2019_11137_MOESM1_ESM.pdf]

SUPPLEMENTARY INFORMATION

Molecular retargeting of antibodies converts immune defense against oncolytic viruses into cancer immunotherapy

Niemann et al.

Supplementary figure 1

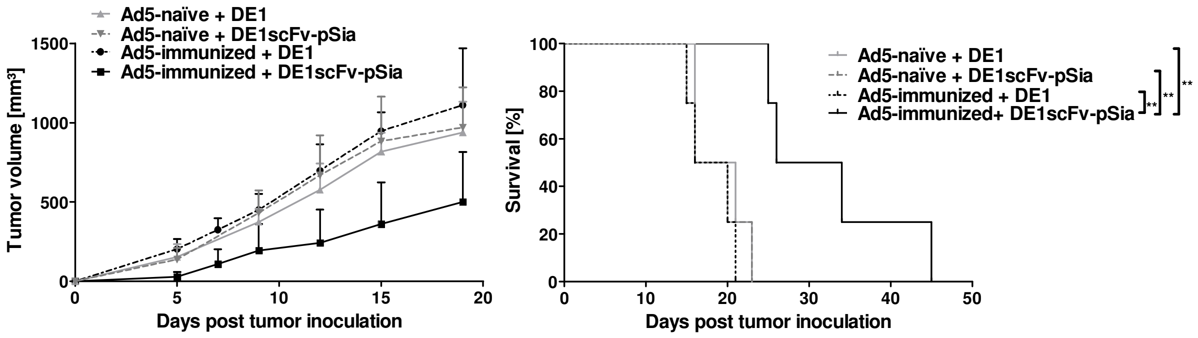

Supplementary figure 1

Tumor growth inhibition by DE1scFv-pSia depends on the presence of antiadenovirus antibodies and requires the retargeting domain.

DE1 and DE1scFv-pSia expressing CMT-pSia cells were generated by transfection of CMT-pSia cells with pT3-based transposon vectors (Addgene) containing either a DE1 expression cassette or a DE1scFv-pSia expression cassette. For stable integration into the cell genome a plasmid expressing the Sleeping-Beauty transposase (SB13, kindly provided by D. Largaespada, Univ. of Minnesota) was added to the transfection mixture using polyethylene-imine (Polyscience) as transfection reagent. Transgene expression and secretion (e.g. DE1 or DE1scFv-pSia expression/secretion) was confirmed via western blot analysis from cell culture supernatants using standard protocols. The obtained CMT-pSia expressing either the Ad5 hexon DE1 domain or the adapter molecule DE1scFv-pSia were then implanted in Ad5-naïve or Ad5-immunized mice to form s.c. tumors. Tumor development and survival was monitored (n=4 in all groups; ms in days: Ad5-naïve + DE1 18.5; Ad5-naïve + DE1scFv-pSia 18; Ad5-immunized + DE1 18; Ad5-immunized + DE1scFv-pSia 30). Log-rank (Mantel-Cox) test was used to calculate survival statistics; $^{**}p \leq 0.01$. Error bars refer to standard deviation (SD).

Supplementary figure 2

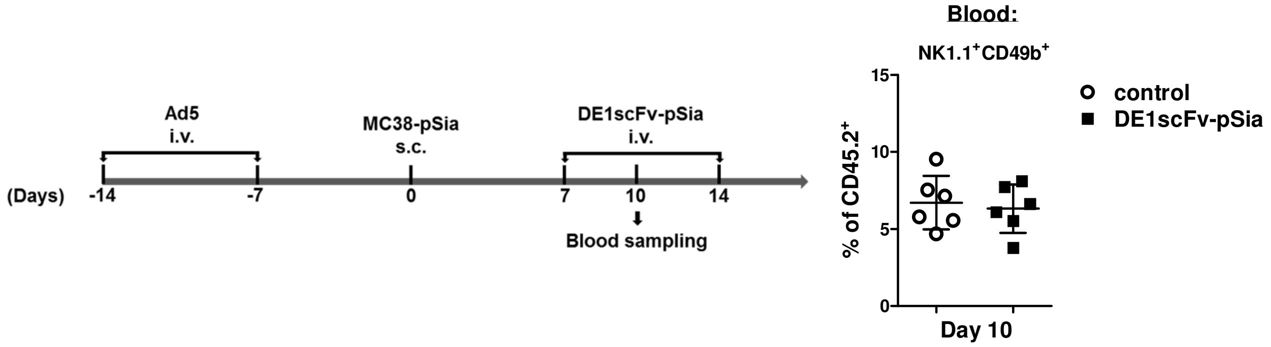

Supplementary figure 2

NK cell frequencies in peripheral blood are not affected by adapter treatment.

The experiment was carried out according to the description in figure 3a. Blood was drawn on day 10 and frequencies of NK cells (NK1.1$^+$CD49b$^+$ cells) within the CD45.2$^+$ leucocyte-subset were determined via flow cytometry (n=6). Error bars refer to standard deviation (SD).

Supplementary figure 3

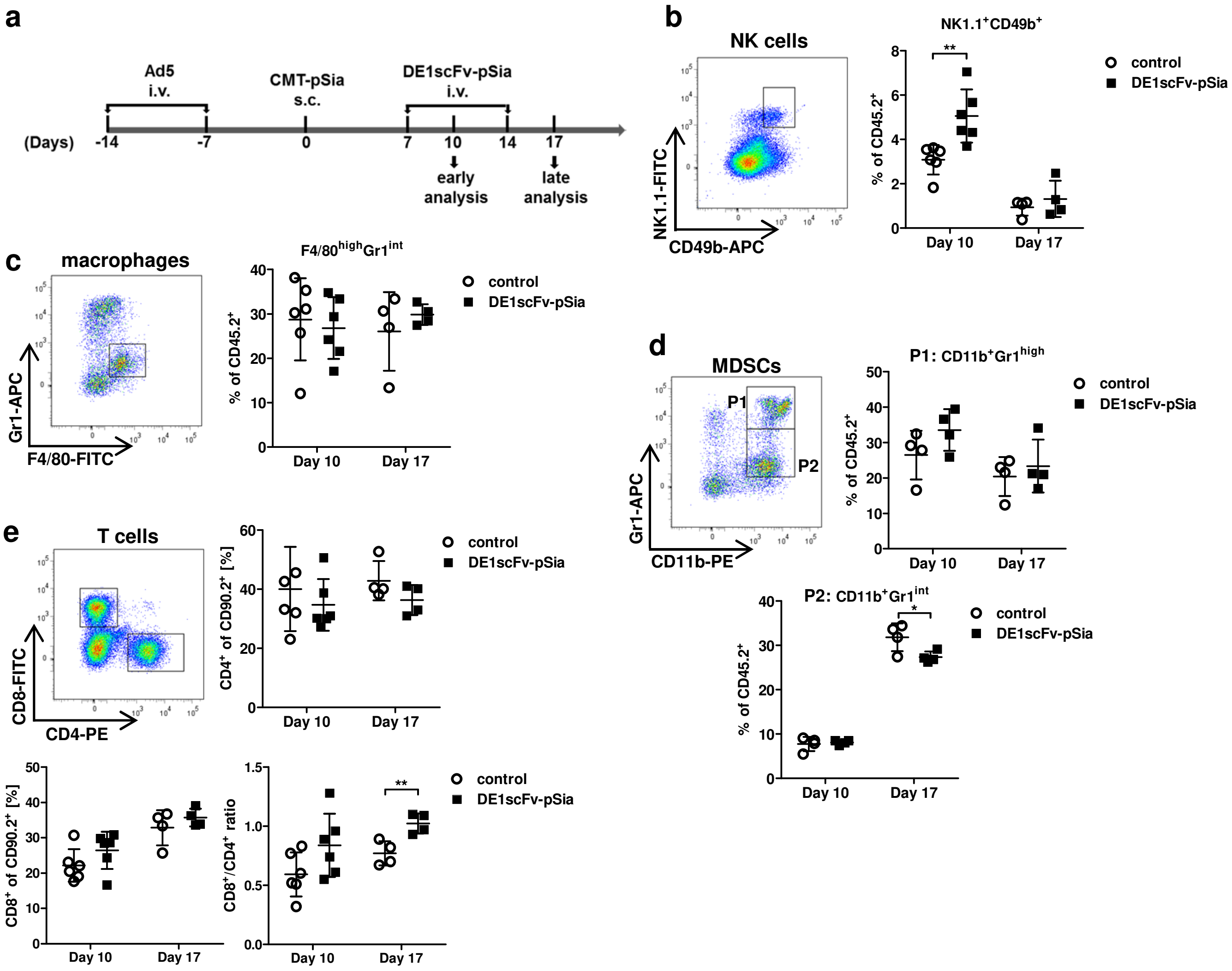

Supplementary Figure 3

Investigations on immune cell infiltrates in CMT-pSia tumors following treatment with DE1scFv-pSia.

a The experiment was carried out according to the description in Figure 2a, CMT-pSia cells were used to establish s.c. tumors. During treatment phase animals were sacrificed at the indicated time points and tumor tissue was examined for infiltration of different leucocyte subsets via flow cytometry. **b-d** Frequencies of NK cells ($NK1.1^{+}CD49b^{+}$), Macrophages ($Gr1^{+}F4/80^{high}$) and myeloide derived suppressor cells (MDSCs; P1: $CD11b^{+}$ $Gr1^{high}$; P2: $CD11b^{+}$ $Gr1^{int}$) were calculated as percentage of CD45.2 positive leucocytes. Group size day 10 n=6 and day 17 n=4 with the following exception: MDSC populations day 10 n=4. **e** Frequencies of $CD4^{+}$ and $CD8^{+}$ T cells were measured as percentage of CD90.2 positive lymphocytes and individual ratios of CD8 to CD4 T cells were calculated. Group size day 10 n=6 and day 17 n=4. Two tailed unpaired t test was used to calculate statistics in B - F: *p≤0.05; **p≤0.01. Error bars refer to standard deviation (SD).

Supplementary figure 4

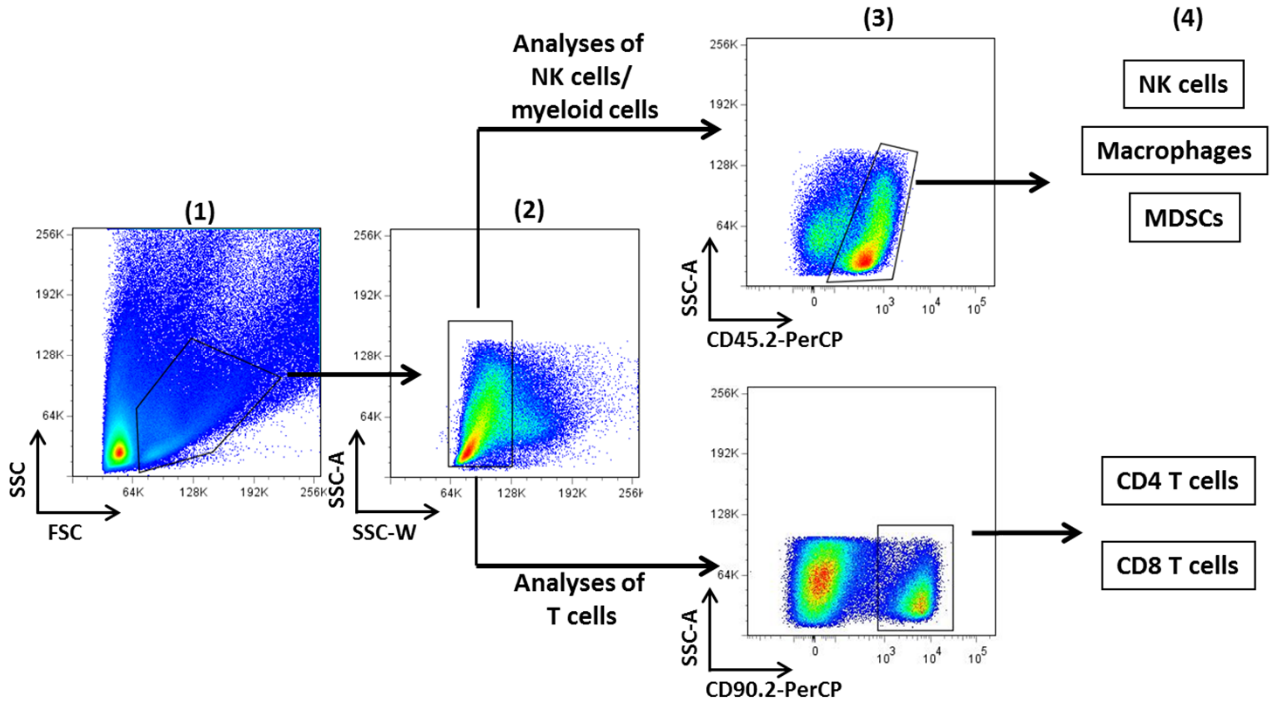

Supplementary Figure 4

Gating strategy to identify immune cell populations via FACS analyses

From left to right: **(1)** The leucocyte population was identified via forward and sideward scatter (FSC/SSC) and **(2)** single cells (identified via SSC-W) were subjected to further analyses : **(3)** CD45.2 cells were identified via staining with a CD45.2-PerCP antibody and CD90.2 expressing lymphocytes were selected via staining with a CD90.2-PerCP antibody . **(4)** Subpopulations such as NK cells and myeloid cells were determined as percentage of CD45.2 positive cells as described exemplarily in the according figures 3b-d; T cell populations were determined as percentage of CD90.2 positive lymphocytes as described exemplarily in Fig. 3e+f.
