# Supplementary material for: Molecular retargeting of antibodies converts immune defense against oncolytic viruses into cancer immunotherapy
Source: Nat Commun. 2019 Jul 19;10:3236. doi: 10.1038/s41467-019-11137-5 (PMC6642145; doi:10.1038/s41467-019-11137-5)
Supplement: Supplementary file 2 — reporting summary [file 41467_2019_11137_MOESM2_ESM.pdf]

nature research

Corresponding author(s): Florian Kuehnel

Last updated by author(s): Jun 11, 2019

Reporting Summary

Nature Research wishes to improve the reproducibility of the work that we publish. This form provides structure for consistency and transparency in reporting. For further information on Nature Research policies, see Authors & Referees and the Editorial Policy Checklist.

Statistics

For all statistical analyses, confirm that the following items are present in the figure legend, table legend, main text, or Methods section.

n/a	Confirmed	
☐	☒	The exact sample size (*n*) for each experimental group/condition, given as a discrete number and unit of measurement
☐	☒	A statement on whether measurements were taken from distinct samples or whether the same sample was measured repeatedly
☐	☒	The statistical test(s) used AND whether they are one- or two-sided<br>*Only common tests should be described solely by name; describe more complex techniques in the Methods section.*
☒	☐	A description of all covariates tested
☒	☐	A description of any assumptions or corrections, such as tests of normality and adjustment for multiple comparisons
☐	☒	A full description of the statistical parameters including central tendency (e.g. means) or other basic estimates (e.g. regression coefficient) AND variation (e.g. standard deviation) or associated estimates of uncertainty (e.g. confidence intervals)
☐	☒	For null hypothesis testing, the test statistic (e.g. *F*, *t*, *r*) with confidence intervals, effect sizes, degrees of freedom and *P* value noted<br>*Give P values as exact values whenever suitable.*
☒	☐	For Bayesian analysis, information on the choice of priors and Markov chain Monte Carlo settings
☒	☐	For hierarchical and complex designs, identification of the appropriate level for tests and full reporting of outcomes
☒	☐	Estimates of effect sizes (e.g. Cohen's *d*, Pearson's *r*), indicating how they were calculated

Our web collection on statistics for biologists contains articles on many of the points above.

Software and code

Policy information about availability of computer code

Data collection	n/a
Data analysis	n/a

For manuscripts utilizing custom algorithms or software that are central to the research but not yet described in published literature, software must be made available to editors/reviewers. We strongly encourage code deposition in a community repository (e.g. GitHub). See the Nature Research guidelines for submitting code & software for further information.

Data

Policy information about availability of data

All manuscripts must include a data availability statement. This statement should provide the following information, where applicable:

- Accession codes, unique identifiers, or web links for publicly available datasets
- A list of figures that have associated raw data
- A description of any restrictions on data availability

All data generated or analysed during this study are available from the authors. The source data underlying all figures are provided as a source data file. A reporting summary has been included. The data availability statement has been included in the manuscript.

Field-specific reporting

Please select the one below that is the best fit for your research. If you are not sure, read the appropriate sections before making your selection.

☒ Life sciences ☐ Behavioural & social sciences ☐ Ecological, evolutionary & environmental sciences

October 2018

Life sciences study design

All studies must disclose on these points even when the disclosure is negative.

Sample size

Sample sizes were chosen according to the basis of previous publications in the immunology field and our own previous experience in pilot experiments. In most of the experiments, 4 - 9 samples per experimental group were considered sufficient to identify significant differences. Exact sample sizes per group have been reported in detail in the figure legends.

Data exclusions

No data have been excluded

Replication

Fig. 1c+d: The experiment has been replicated three or more times with multiple replicates per group showing comparable results.
Fig 1 e: the experiment has been replicated three or more times with similar results.
Fig 2a-d: the experiment as shown has been performed once with multiple replicates per group. Results of selected groups included in figure 2b (ad5-immunized: Ctrl and DE1scFv-pSia) have been replicated in a second independent experiment with similar results.
Fig 2e-f: the experiment has been performed twice with similar results (statistically significant).
Fig. 3: the experiments have been performed once with multiple replicates per group as indicated in the figure legend.
Fig. 4: the experiments have been performed once with multiple replicates per group as indicated in the figure legend. A second independent experiment has confirmed the results of the Ctrl and DE1scFv-pSia groups with similar results (statistic significance).
Fig. 5: the experiments have been performed once with multiple replicates per group. The results of the NaCl, hTert-Ad i.t. and hTert-Ad i.t. + DE1scFv-pSia groups have been confirmed in a second independent experiment (statistic significance).
Fig. 6: the experiments have been performed once with multiple replicates per group as indicated in the figure legend. A second independent experiment containing the NaCl, hTert-Ad, and hTert-Ad + DE1scFv-pSia groups has been performed with similar results (statistic significance)

Randomization

Groups of mice were assembled to reflect a spectrum of different tumor sizes and a comparable 'total' tumor mass. Assignment of these groups to experimental treatments was done in a randomized fashion.

Blinding

For in vivo experiments scientists were blinded to the experimental group by labeling cages by a group code, and not by the treatment scheme.

Reporting for specific materials, systems and methods

We require information from authors about some types of materials, experimental systems and methods used in many studies. Here, indicate whether each material, system or method listed is relevant to your study. If you are not sure if a list item applies to your research, read the appropriate section before selecting a response.

Materials & experimental systems

n/a	Involved in the study
☐	☒ Antibodies
☐	☒ Eukaryotic cell lines
☒	☐ Palaeontology
☐	☒ Animals and other organisms
☒	☐ Human research participants
☒	☐ Clinical data

Methods

n/a	Involved in the study
☒	☐ ChIP-seq
☐	☒ Flow cytometry
☒	☐ MRI-based neuroimaging

Antibodies

Antibodies used

antigen (clone no.): CD90.2-PerCP (30-H12), CD45.2-PerCP (104), CD8-FITC (53-6.7), CD8-PE (53-6.7), CD4-PE (GK1.5), CD4-APC (GK1.5), Gr1-APC (RB6-8C5), CD11b-PE (M1/70), F4/80-FITC (BM8), IFNγ-APC (XMG1.2), NK1.1-PE (PK136), NK1.1-FITC (PK136) CD49b-FITC (DX5), CD49b-APC (DX5), CD107a-PE (1D4B), c-myc (9E10), anti-polySia (mAb735), CD8 (116-13.1), NK1.1 (PK136), PD-1 (RMP1-14). The following antibodies were used as isotype controls: CD107a/ F4/80/ CD8: rat IgG2a (RTK2758); NK1.1/ CD45.2, mouse IgG2a (MOPC-173); CD49b: rat IgM (RTK2118); CD11b/ CD4/ Gr-1/ CD90.2: rat IgG2b (RTK4530); IFNy: rat IgG1 (RTK2071). Antibodies were diluted 1:100

Validation

All antibodies were validated by their specific staining patterns by FACS analysis and compared to known patterns in the literature and those provided by the manufacturer.

Eukaryotic cell lines

Policy information about cell lines

Cell line source(s)

The cell lines HEK293 (CRL-1573), 293T (CRL-3216), PhoenixAMPHO (CRL-3213), Panc01 (CRL-1469), TE671 (CRL-8805), IMR32 (CCL-127), and B16F10 (CRL-6475) were obtained from ATCC. CMT64 cells (10032301) were obtained from the

	European Collection of Cell Cultures (ECACC) and MC38 cells were kindly provided by Michael Neumaier, Univ. of Mannheim, Germany
Authentication	After purchase, cell lines were not authenticated again, but were verified for characteristic growth patterns according to the information available by the cell bank. Cell lines were used at early passage to make transgenic, polySia expressing variants as described in the manuscript. Transgenic variants were routinely checked by FACS for cell surface polySia to confirm transgene expression, and were eventually re-selected with the appropriate selection marker. MC-38 cells were authenticated by the mutation-specific CD8 T cell response against the neoantigen Adpgk-R304M
Mycoplasma contamination	All cell lines were routinely tested for mycoplasm contamination
Commonly misidentified lines (See ICLAC register)	TE671 are included in the ICLAC register. As correctly stated in the paper, this cell line is a rhabdomyosarcoma cell line.

Animals and other organisms

Policy information about studies involving animals; ARRIVE guidelines recommended for reporting animal research

Laboratory animals	C57BL/6, female and male, age 6 weeks
Wild animals	*Provide details on animals observed in or captured in the field; report species, sex and age where possible. Describe how animals were caught and transported and what happened to captive animals after the study (if killed, explain why and describe method; if released, say where and when) OR state that the study did not involve wild animals.*
Field-collected samples	*For laboratory work with field-collected samples, describe all relevant parameters such as housing, maintenance, temperature, photoperiod and end-of-experiment protocol OR state that the study did not involve samples collected from the field.*
Ethics oversight	The planned study design has been controlled by the local institutional organisation responsible for animal care (central animal laboratory ZTL; MHH) and has been finally approved by the regional legal authorities responsible for animal welfare (LAVES, Oldenburg, Germany)

Note that full information on the approval of the study protocol must also be provided in the manuscript.

Flow Cytometry

Plots

Confirm that:

☒ The axis labels state the marker and fluorochrome used (e.g. CD4-FITC).

☒ The axis scales are clearly visible. Include numbers along axes only for bottom left plot of group (a 'group' is an analysis of identical markers).

☒ All plots are contour plots with outliers or pseudocolor plots.

☒ A numerical value for number of cells or percentage (with statistics) is provided.

Methodology

Sample preparation	Tumor tissue was digested in RPMI 1640 medium (Gibco) containing DNAse (60 µg/ml), hyaluronidase (0.2 µl/ml), Collagenase IA and IV (0.2 mg/ml each) for 30 min at 37°C. Required enzymes were obtained from Sigma. Cell solutions were subsequently washed with RPMI and filtered using a 40 µm cell strainer to obtain single cell preparations.<br>Splenocytes were released from spleens by passing through a 40 µm cell strainer and washed with RPMI 1640 (Gibco). Erythrocytes were lysed by adding 1x EBC Lysis Buffer (BioLegend) for 5 min at 4°C and cells were subsequently washed with RPMI 1640. Cells were resuspended in RPMI 1640 medium supplemented with 2 % FCS, 100 U/mL streptomycin, 100 mg/mL penicillin, 1 % MEM with non-essential amino acids (100x solution, Gibco) ß-mercaptoethanol (50 µM, Sigma) and sodium pyruvate (1 mM, Gibco) and kept at 37°C and 5 % CO2 overnight during peptide stimulation.<br>Erythrocytes were removed from blood samples by adding 1x EBC Lysis Buffer (BioLegend) and incubated for 3 min at 4°C. Cells were subsequently washed with RPMI 1640 (Gibco).
Instrument	Multicolor FACS analysis was perfomed using a BD FACS Canto II device
Software	FACS data were obtained with BD FACS software (FACS-Diva), all data analysis has been performed using the flow cytometry analysis program FlowJo (Treestar)
Cell population abundance	2 x 10E5 - 2 x 10E6 cells were analysed per sample.
Gating strategy	For the data analysis, FSC-A/SSC-A gating was performed for cell size and exclusion of<br>debris and identification of leukocytes. In addition, FSC-A/SSC-W gating was performed for the exclusion of cell doublets. The subsequent gating strategy for defined subpopulations is described in the figure legends and is visualized in a supplementary figure. Additionally, a representative plot showing the positive gate is included in each figure.

☒ Tick this box to confirm that a figure exemplifying the gating strategy is provided in the Supplementary Information.
